# Simulation-based medical training for paediatric residents in Italy: a nationwide survey

**DOI:** 10.1186/s12909-019-1581-3

**Published:** 2019-05-22

**Authors:** Marco Binotti, Giulia Genoni, Stefano Rizzollo, Marco De Luca, Luca Carenzo, Alice Monzani, Pier Luigi Ingrassia

**Affiliations:** 1 Neonatal and Paediatric Intensive Care Unit, Maggiore della Carità Hospital, Novara, Italy; 2 SIMNOVA, Interdepartmental Centre for Innovative Didactics and Simulation in Medicine and Health Professions, University of Piemonte Orientale, Novara, Italy; 3 Division of Paediatrics, Department of Health Sciences, University of Piemonte Orientale, Via Solaroli 17, 28100 Novara, Italy; 4 SIMMeyer, Anna Meyer Children’s University Hospital, Florence, Italy

**Keywords:** Simulation, Simulation-based medical training, Survey, Paediatrics, Residents

## Abstract

**Background:**

A prompt start to an appropriate neonatal and paediatric resuscitation is critical to reduce mortality and morbidity. However, residents are rarely exposed to real emergency situations. Simulation-based medical training (SBMT) offers the opportunity to improve medical and non-technical skills in a controlled setting. This survey describes the availability and current use of SBMT by paediatric residents in Italy with the purpose of understanding residents’ expectations regarding neonatal and paediatric emergency training, and identifying gaps and potential areas for future implementation.

**Methods:**

A survey was developed and distributed to Italian residents. SBMT was defined as any kind of training with a mannequin in a contextualised clinically realistic scenario.

**Results:**

The response rate was 14.4%, covering the 71% of Italian paediatric residency programmes. Among them, 88% stated that Out of the 274 residents, 88% stated that they received less than 5 h of SBMT during the past training year, with 66% not participating in any kind of simulation activity. In 62% of the programmes no simulation training facility was available to residents. Among those who received SBMT, 46% used it for procedures and skills, 30% for clinical scenarios, but only 24% of them reported a regular use for debriefing. Of the overall respondents, 93% were interested in receiving SBMT to improve decision-making abilities in complex medical situations, to improve technical/procedural skills, and to improve overall competency in neonatal and paediatric emergencies, including non-technical skills. The main barriers to the implementation of SBMT programmes in Italian paediatric residencies were: the lack of experts (57%), the lack of support from the school director (56%), the lack of organisation in planning simulation centre courses (42%) and the lack of teaching materials (42%).

**Conclusions:**

This survey shows the scarce use of SBMT during paediatric training programmes in Italy and points out the main limitations to its diffusion. This is a call to action to develop organised SBMT during paediatric residency programs, to train qualified personnel, and to improve the quality of education and care in this field.

**Electronic supplementary material:**

The online version of this article (10.1186/s12909-019-1581-3) contains supplementary material, which is available to authorized users.

## Background

Simulation-based medical training (SBMT) has been defined as the artificial representation of a complex real-world process with sufficient fidelity with the aim to facilitate learning through immersion, reflection, feedback, and practice minus the risks inherent in a similar real-life experience [1]. It may be based on the use of mannequins, partial task trainers, virtual reality, or trained actors, as an alternative to real patients, allowing for the creation of realistic but well-controlled clinical settings that simulate real-life patient care. The efficacy of SBMT as teaching method for paediatric education has been assessed [2, 3]. Many studies have shown that paediatric residents’ resuscitation skills are inadequate, with little improvement during residency [4, 5]. These findings suggest that it is insufficient to rely on direct clinical exposure alone to achieve the requirements for emergency care skills [6]. Conversely, SBMT has been associated with improvement in key measures of quality life support and progressive acquisition of resuscitation skills during paediatric training [5]. Furthermore, SBMT is an effective method to teach non-technical and behavioural skills such as teamwork, leadership, communication and role clarity [7]. Moreover, some paediatric milestone competencies are difficult to assess using traditional methodologies. SBMT meets the needs of programme directors to acquire measurable learning outcomes on their residents’ performances [8]. For these reasons, the use of SBMT in paediatric residencies has grown over the past decade. In U.S. and Canadian paediatric emergency medicine, SBMT was being provided by 97% of residency programmes in the 2011–2012 academic year [9]. Recent surveys showed that SBMT is used by more than 90% of U.S., Canadian and English-language-based emergency medicine residency programmes, even though a considerable variability in the accreditation and certification, and frequency and timing of SBMT has been highlighted [10, 11].

Conversely, up until recently, the use of SBMT was scarce in European paediatric residencies. In 2009, only 3 Swiss paediatric healthcare institutions used SBMT [12], rising to 20/30 (66.6%) of paediatric hospitals and healthcare departments offering SBMT in 2015 [13].

The aim of this study is to describe the current use of simulation in paediatric residency programmes in Italy, in order to understand the expectations of residents with regard to neonatal and paediatric emergency training, as well as to identify gaps and potential areas for future implementation.

## Methods

### Survey development and content

A 40-item survey was developed by simulation experts from two Italian simulation centres, SIMNOVA (Novara) and SimMeyer (Florence). The questionnaire was composed of multiple choice questions and questions in which participants rated their agreement on a 10-point Likert scale (1 = strongly disagree – 10 = strongly agree) (Additional file 1).

For the purpose of this survey, SBMT was defined as the use of a high fidelity neonatal or paediatric simulators which can reproduce different clinical symptoms and signs in response to therapeutic interventions performed; the reconstruction of clinical cases in a simulation room which most accurately reproduces the environment subject of the simulation (delivery room, paediatric emergency room, neonatal or paediatric intensive care, etc.); a phase of debriefing following the clinical case. The survey included questions evaluating: the degree of interest toward simulation, the current use of SBMT, the availability of facilities and support resources in Italian paediatric residency programs, the benefits of simulation perceived by paediatric residents and potential barriers limiting its current use.

### Survey dissemination

The survey was disseminated to all the 35 Italian paediatrics residency programme directors, asking them to distribute it among their respective residents. The survey was also available on a web-based survey tool (www.surveymonkey.com; SurveyMonkey, Inc., Palo Alto, CA) for 12 months from the 1st April 2016 to the 1st April 2017. Moreover, it was directly disseminated by the National Observatory of Paediatric Residents (ONSP) via its official website page and three consecutive newsletters. In addition, a paper copy of the survey was delivered to the participants of two national events: the 2016 ONSP National Congress in Bologna and the Paediatric Simulation Experience 2016 in Novara. It was possible to answer the web-based survey only once, and in the questionnaire’s written instructions, respondents were asked to answer only once. The participation in the survey was voluntary, anonymous, and independent. Confidentiality of information was ensured and no financial incentive to participate in the study was offered. Responses to the survey were presented in aggregate form. Completion of the study questionnaire implied participant consent. The Ethics Committee of ‘Maggiore della Carità’ Hospital (Novara, Italy) deemed that a formal ethical approval was not required as the study was a survey. Results are presented as number (percentage) for discrete variables or as median and interquartile range (IQR) for continuous variables, as appropriate.

## Results

### Study sample

A total of 274 questionnaires were returned, out of 1900 Italian paediatric residents (14.4%). Respondents were from 25 out of 35 Italian paediatric residencies (71%), with a median of 7 respondents per residency (IQR 5–9), from different geographical areas: Northern Italy (47%), Central Italy (22%) and Southern Italy (31%). Respondents were attending the first, second, third, fourth and fifth years of residency programmes in 12, 22, 21, 26 and 19% of cases, respectively.

### Simulation exposure

**Fig. 1:**
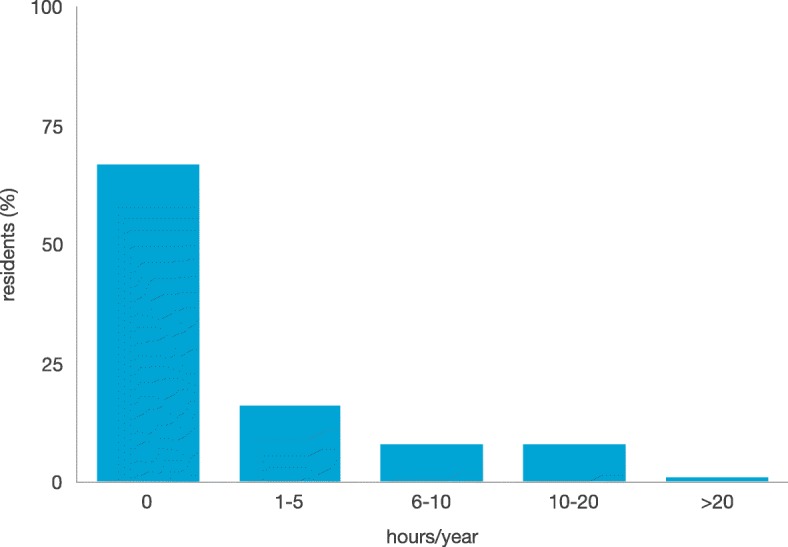
Simulation activity of Italian paediatric residents in the 2015-2016 academic year. The graph shows the percentage of Italian paediatric residents respectively spending 0, 1–5, 6–10, 10–20, > 20 h in simulation activities during 2015–2016

In the 2015–2016 academic year, 88% of respondents spent less than 5 h in SBMT, with approximately 66% not participating in any kind of simulation activity (Fig. 1). The 29% of respondents reported that their residency programmes offered SBMT as a support for teaching neonatal and paediatric emergency care. Eighteen percent of respondents believed that in their residency there was a plan to develop SBMT in the next 1–2 years. Twenty-two percent had attended simulation courses organised by other schools. The reported SBMT programmes were focused on elements of procedural training (46%) and, to a lesser extent, on the creation and development of scenarios (30%), and debriefing (24%). SBMT was used for the assessment of resuscitation skills in 15% of residents. Only 5% of respondents had been trained in teaching the use of simulation and only 1% had done research in this field.

Table 1 shows the method used to teach technical skills during paediatric residency. Compared to bedside practice and other teaching methodologies like frontal lessons, SBMT was the preferred method used to teach chest compressions (45%), cardioversion/defibrillation (36%), endotracheal intubation (33%), difficult airway management (21%) and intraosseous access (30%).

**Table 1:** Technical skills’ teaching methods reported by the 274 respondents to the survey

	SBMT	Bedside	Other methods	Not taught or not evaluated
Mask ventilation % (*n*)	29% (79)	33% (90)	11% (31)	27% (75)
Endotracheal intubation % (*n*)	33% (90)	19% (52)	6% (17)	42% (115)
Difficult airway management % (*n*)	21% (58)	7% (20)	5% (13)	67% (183)
Chest compressions % (*n*)	45% (124)	17% (47)	6% (16)	32% (87)
Cardioversion/defibrillation % (*n*)	36% (99)	3% (8)	3% (8)	58% (159)
Central venous access % (*n*)	7% (18)	24% (67)	4% (10)	65% (179)
Umbilical venous access % (*n*)	9% (25)	54% (148)	7% (19)	30% (82)
Intraosseous access % (*n*)	30% (82)	7% (19)	3% (8)	60% (165)
Lumbar puncture % (*n*)	7% (19)	39% (107)	16% (43)	38% (105)
Chest tube placement % (*n*)	8% (23)	12% (32)	4% (11)	76% (208)

### Simulation facilities and resources

In 62% of residency programmes, there was neither a simulation-training centre nor an affiliation with another institution’s simulation centre. In schools with or affiliated with simulation facilities, the laboratory was easily accessible, either located in the same building (74%) or in another building within a 5–10-min walking distance (19%) from the department, or reachable by public transport or car (7%). In 66% of cases, existing laboratories were made up of just a simulation room. In the remaining cases, the simulation centre also consisted of a control room (38%), a debriefing room (32%), multifunctional rooms with audio-visual capabilities (23%) and other rooms (34%). However, even where a laboratory was available, a lack of support personnel was reported in 53% of questionnaires. Where present, the staff consisted of healthcare professionals such as doctors, nurses or postgraduates who coordinated simulation activities (69%), simulation instructors (18%) and other personnel (13%). Regarding the available equipment, 24% had low-fidelity mannequins, 22% used high-fidelity neonatal and paediatric mannequins, and 54% did not know. Regarding funding sources for simulation, 12% reported that the funds came from the university, 28% answered that there are no available funds dedicated to SBMT and 60% did not know where funding came from.

#### Perceived benefits of SBMT

The perceived preparation of residents to effectively manage paediatric and neonatal emergencies expressed as median (IQR) was 4 (3–6) and 5 (4–7), respectively. We found no difference in the confidence to manage neither a paediatric nor a neonatal emergency according to the year of residency, namely the median value (IQR) was 4 (3–5) in residents of the I-II year of residency vs 4 (3–6) in residents of the III-IV-V year for the management of paediatric emergencies (*p* = 0.737), and 4 (3–5) in residents of the I-II year of residency vs 5 (4–6) in residents of the III-IV-V year for the management of neonatal emergencies (*p* = 0.202). Similarly, no differences were found according to the degree of exposure to previous SBMT experiences: for paediatric emergencies, 5 (4–6) in those who previously attended one SBMT experience vs. 5 (4–7) in those who never had SBMT (*p* = 0.803); for neonatal emergencies, 5 (3–6) in those who previously attended one SBMT experience vs. 5 (3–6) in those who never had SBMT (*p* = 0.901). The interest for SBMT was high (93%). When asked about perceived benefits of SBMT, 99.6% of respondents stated that it is helpful in improving decision-making abilities in complex situations; 99.3% agreed that it is helpful in improving technical/procedural skills; 99.6% agreed that, overall, it is an effective tool to improve neonatal and paediatric emergency medicine competence; and, finally, 97.4% agreed that it is a valuable tool for non-technical skills such as leadership, communication and team management, without differences according to having or not experienced SBMT previously.

#### Barriers to the use of SBMT

The main barriers to the implementation of SBMT programmes in Italian paediatric residencies were: the lack of experts (57%), the lack of support from the school director (56%), the lack of organisation in planning simulation courses (42%), and the lack of teaching materials (42%).

The main limits to external course participation were: costs related to external training, including travel and accommodation (73%), lack of time (54%), and the unavailability of SBMT courses nearby (29%).

## Discussion

Despite the low response rate, this survey represents a realistic snapshot of SBMT in Italy, involving 71% of Italian paediatric residency programmes and including paediatric residents from the whole country. Our survey reveals the scarce use of SBMT during paediatric training in Italy. Two thirds of respondents did not participate in any kind of simulation activity during the previous academic year, and the majority of programmes did not implement neonatal and paediatric emergency training with SBMT. These data contrast with the current situation in other developed countries, such as the U.S., Canada, Switzerland, Germany, Austria, and South Tyrol, where SBMT has been implemented into educational curricula [14] and offered by the majority of paediatric residency programmes, even though great differences between various countries in terms of SBMT’s accreditation, frequency and timing are still reported [9–11, 13, 15].

Increasing evidence suggests that SBMT improves healthcare education, practice and patient safety, allowing learners to achieve competence without putting patients at risk. The literature suggests that simulation in medical education improves both technical and non-technical skills [3, 5, 7, 16]. Regarding technical skills, the learning of some procedures is a crucial component of paediatric education, and it represents an accreditation requirement for paediatric training programmes in Canada and Australia [17, 18]. Furthermore, in 2007, the U.S. Residency Review Committee published a list of procedures and skills in which residents should have “sufficient” experience [19]. In Italy, every paediatric residency has its own training objectives, including a list of procedural skills that residents need to acquire. In this study, we show that SBMT is mostly used for this purpose. However, as shown by others and us, training for some important neonatal and paediatric emergency care procedures (like positioning of thoracic drainage and central venous access or management of difficult airways), is often not provided. As a result, a large percentage of residents fail to achieve procedural skills competence [20]. This highlights a weakness in Italian paediatric education and should be considered as a starting point to improve the quality of training programmes and, finally, the expertise of future paediatricians. From this perspective, simulation is an ideal method, on the one hand, to learn these skills by integrating the possibility of direct observation, frequent practice and feedback, and, on the other hand, to objectively evaluate the achievement of these competences. Studies using simulation task trainers to teach paediatric residents procedures, such as central venous catheter, chest tube insertion and endotracheal intubation, showed improved performance, demonstrating that simulation is an effective educational tool [21–24]. Furthermore, studies have shown that the majority of paediatric residents have an insufficient knowledge and experience in the care of critically ill children due to their low exposure to such conditions [6, 25]. It has been previously reported that simulation can improve paediatric residents’ performances during high-risk situations like cardiopulmonary arrest and paediatric trauma [26–29].

Another crucial component of SBMT is the acquisition and improvement of non-technical and behavioural skills, such as teamwork, leadership, communication and role clarity [7]. However, from this survey, this aspect seems of secondary importance in Italian paediatric education, and this highlights another important gap that urgently needs to be addressed. Indeed, some “high-impact” conditions, like neonatal and paediatric resuscitation, besides being rare conditions, usually involve multidisciplinary teams, like paediatricians and anaesthesiologists, and improving communication and teamwork may lead to a better patient outcome.

In our survey, we found no differences in the confidence to manage a paediatric/neonatal emergency neither according to the year of residency nor according to the previous exposure to SBMT experience. The possible explanation of these results may be on the one hand that the confidence in managing an emergency probably does not increase along the residency program, due to the known low exposure of residents to real emergencies, and on the other hand that the overall exposure of respondents to SBMT is too low to acquire confidence in the management of emergencies.

Despite the low diffusion of SBMT in Italy, paediatric residents show an extremely high interest in acquiring basic knowledge and skills through SBMT. They perceive its potential key benefits to improve decision-making abilities in complex medical situations and to learn technical/procedural and non-technical skills. The apparent discrepancy between the high perceived benefit of SBMT and actual resident exposure to SBMT may be explained by what residents know from literature data or from other residents’ experiences, and by what they have personally experienced during courses like Paediatric Simulation Experience, outside their residency programmes [30]. Italian residents identified the lack of paediatric simulation educators, the lack of support from the school director and the lack of organisation in planning simulation courses and teaching materials as the main barriers for the development of SBMT. To counteract these problems, along with the growing interest in SBMT, some Italian simulation centres offer courses for residents like Paediatric Simulation Experience developed by SIMNOVA (Novara) and SimMeyer (Florence) [30]. However, there are some limitations to external course participation, such as cost and distance. In Switzerland, during the last few years, a significant surge in the use of SBMT has been shown, increasing from 3 institutions in 2009 to 20 out of 30 institutions in 2015 [13]. The majority of units offered SBMT in an in-situ setting, and this could limit costs relating to the creation of a simulation centre in those hospitals that do not already have one. Furthermore, more than one residency school can aggregate in a single simulation centre to limit costs and the need for personnel.

Implementing SBMT would be an useful tool to allow residents learning in a safe way. As previously argued by Savoldelli et al. [31], SBMT may represent the way to move away from a culture of silence and blame when facing an error to a new culture of safety, where errors are disclosed and analysed in the debriefing. In particular, Savoldelli et al. highlighted the crucial role of performing such a change of culture in young subjects, because it is likely that residents experiencing high-fidelity simulation, will probably continue SBMT when they become staff. Moreover, they may also encourage peers to participate to SBMT if their experiences in simulation are positive [31].

It is widely recognized that costs represent one of the main barriers to the implementation of SBMT [32], as the investment required for the development and maintenance of a simulation program can be sometimes prohibitive. Our data demonstrated that the lack of equipment and personnel are perceived as substantial limitations. One potential solution may be to promote the creation of regional simulation centres as they would allow sharing of equipment and faculty resources. As previously reported, regionalization of accredited programs would be a strategy to promote wider access to high-quality training [33–35]. A second possible solution is the use of in situ simulation, which may represent a cost-sparing but highly effective alternative [36, 37].

In conclusion, this survey represents the first attempt to provide a baseline assessment of the interest among Italian pediatric residents in SBMT and to learn of their opinions about the unmet needs in this field. Starting from the results driven from the current survey, we have developed a poll to be surveyed to the Directors, to assess also their point of view and perception of the current Italian situation, to be integrated to the residents’ counterpart.

## Limitations

Our study had several limitations. Despite efforts to boost participation, the sample size was quite small. However, this study covered 71% of training programmes in Italy, equally distributed throughout the country, and this is the first comprehensive description of the current use of simulation in paediatric residencies in Italy. In addition, a selection bias cannot be excluded, as residents who were more interested in the topic were probably more likely to reply. Another limit of this study is that about one third of the respondents were from the first two (of five) years of residency. However, in Italy, SBMT is not scheduled at a fixed point in time during residency programmes. Moreover, even if our focus was on high-fidelity simulation, medium and low fidelity mannequins were included limited to technical skills. Finally, even though the questionnaire was internally validated based on the consensus of the experts from two centres, it was never tested for reliability.

## Conclusions

In conclusion, this survey reveals the scarce use of SBMT by Italian paediatric residency programmes and points out the main barriers that prevent SBMT diffusion. This is a call to action to develop organised SBMT during paediatric residency programmes, to train qualified personnel and to carry out research in this field in order to improve the quality of education and care.

## Additional files

Additional file 1: Survey on the use of simulation in neonatal and paediatric emergency care. (DOCX 103 kb)

## Abbreviations

IQR: interquartile range
SBMT: simulation-based medical training
