# Supplementary material for: Simulation-based medical training for paediatric residents in Italy: a nationwide survey
Source: BMC Med Educ. 2019 May 22;19:161. doi: 10.1186/s12909-019-1581-3 (PMC6529987; doi:10.1186/s12909-019-1581-3)
Supplement: Supplementary file 1 — Survey on the use of simulation in neonatal and paediatric emergency care. (DOCX 103 kb) [file 12909_2019_1581_MOESM1_ESM.docx]

**Additional file 1: SURVEY ON THE USE OF SIMULATION IN NEONATAL AND PEDIATRIC EMERGENCY CARE**

***Welcome. We kindly ask you to carefully read this brief introduction before filling out the survey.***

The aim of this investigation is to evaluate the current use of simulation in neonatal and pediatric emergency training in Italian postgraduate schools.

Response to this survey implies consent to participate in the study.

The questionnaire is composed of **40** questions and will take about 10 minutes to complete.

For the purpose of this survey, simulation is defined as a didactic method which includes:

- The use of a high fidelity neonatal or pediatric simulators which can reproduce different clinical symptoms and signs in response to therapeutic interventions performed;
- The reconstruction of clinical cases in a simulation room which most accurately reproduces the environment subject of the simulation (delivery room, pediatric emergency room, neonatal or pediatric intensive care, etc.);
- a phase of debriefing following the clinical case.

**Basic or advanced traditional neonatal or pediatric resuscitation courses (NLS, PBLS, EPLS, PALS, etc.) are not considered simulation in this survey.**

**QUESTIONNAIRE**

1. Indicate the geographical position of your school:

- Northern Italy
- Central Italy
- Southern Italy

1. What area of pediatrics are you most interested in?

- Pediatric emergency care
- Neonatology
- Other (please specify)

1. What year of residency are you now enrolled in?

| I | II | III | IV | V |
| --- | --- | --- | --- | --- |

1. In addition to Pediatrics, do you have other specialties?

…………………………………………………………………………………………………………

…………………………………………………………………………………………………………

1. On a scale of 1 to 10, express your judgment on the preparation provided by your residency to manage a **pediatric** urgent/emergency care situation,

| 1 | 2 | 3 | 4 | 5 | 6 | 7 | 8 | 9 | 10 |
| --- | --- | --- | --- | --- | --- | --- | --- | --- | --- |

1. On a scale of 1 to 10, express your judgment on the preparation provided by your residency to manage a **neonatal** urgent/emergency care situation.

| 1 | 2 | 3 | 4 | 5 | 6 | 7 | 8 | 9 | 10 |
| --- | --- | --- | --- | --- | --- | --- | --- | --- | --- |

1. Is simulation used in emergency neonatal and pediatric training at your residency?
   - Yes
   - No
2. If your residency does not currently provide simulation-based training, is there a plan to develop such a programme in the next 1-2 years?
   - Yes
   - No
   - Maybe
   - Don’t know

*Indicate your level of agreement with the following statements.*

1. I would like to see more resources invested in simulation because it represents an important didactic tool for decision-making in complex medical situations.
   - Strongly disagree
   - Disagree
   - Neither agree nor disagree
   - Agree
   - Strongly agree
2. I would like to see more resources invested in simulation because I believe it is useful for teaching technical skills for procedures rarely performed in clinical practice.
   - Strongly disagree
   - Disagree
   - Neither agree or disagree
   - Agree
   - Strongly agree
3. I would like to see more resources invested in simulation because I believe it is an important tool to improve non-technical skills (team communication, team management, leadership, etc.)
   - Strongly disagree
   - Disagree
   - Neither agree nor disagree
   - Agree
   - Strongly agree
4. I would like to see more resources invested in simulation because I believe it is an important tool to improve overall competence in neonatal and pediatric emergency care.
   - Strongly disagree
   - Disagree
   - Neither agree nor disagree
   - Agree
   - Strongly agree
5. What factors do you believe impede the development of a simulation-based training programme in your residency? *(indicate all that apply)*
   - Lack of support from the school director
   - Lack of experts in simulation-based training
   - Lack of time available to simulation-based training experts
   - Lack of personnel interested in simulation-based training
   - Lack of organization in planning courses at simulation centres or in the use of mannequins
   - Lack of materials
   - Lack of space dedicated to simulation
   - Lack of support personnel (es. technical personnel competent to use the mannequins, secretary, etc.)
   - Presence of other training programmes
   - Other (please specify)

…………………………………………………………………………………………………

1. Have you ever participated in a simulation course organized by another department (Anesthesia and Resuscitation, Gynecology, etc.)?
   - Yes
   - No
2. Have you been trained to teach others (other postgraduates, medical students, undergraduate students in healthcare professions) on the use of simulation?
   - Yes
   - No
3. Are you interested in learning the basics of simulation as a didactic method?
   - Yes
   - No
4. Have you received training at your residency in the following areas of simulation?

*(indicate all which apply)*

- - Debriefing
  - Creation and development of scenarios
  - Procedural training
  - Other (please specify)
  - Simulation-based courses are not available.

1. Have you received simulation-based training at your residency on psychosocial elements (communication of bad news, etc.)?
   - Yes
   - No
   - Not sure
2. In the last year, how many hours of your training were dedicated to simulation?

o 0

- 1-5

o 6-10

o 11-15

o 16-20

o >20

1. Is simulation used at your residency to assess competence to perform specific procedures?
   - Yes
   - No
   - Not sure
2. Is simulation used at your residency to evaluate resuscitation competence?
   - Yes
   - No
   - Not sure
3. Do you conduct research on simulation?
   - Yes
   - No
4. Does your school have a simulation laboratory or is it affiliated with a simulation facility at another institution?
   - Yes
   - No
   - Not sure
5. If yes, where is the simulation laboratory located?
   - In the same building as my department
   - In another building within 5-10 minutes walking distance
   - In another building accessible by public transport or car
   - Other (please specify)
6. The simulation laboratory is composed of the following areas: *(indicate all which apply)*
   - Simulation room
   - Control room
   - Debriefing room
   - Videotaping room
   - Other (please specify)
7. What simulators are available in your school/university/simulation laboratory?
   - High fidelity neonatal mannequins (Sim NewB, Newborn HAL, etc.)
   - High fidelity pediatric mannequins (SimBaby, Meti Pediatric simulator, etc.)
   - High fidelity adult mannequins (Meti HPS, SimMan, etc.)
   - High fidelity obstetric mannequins (Noelle, etc.)
   - Static mannequins (lacking functions such as respiratory and cardiac sounds, palpable pulses, generation of heart rhythm, etc.)
   - Other (please specify)

- Not sure which simulators are available

1. What support personnel are present in your simulation laboratory to improve its management?
   - Simulation instructors
   - Technical personnel (to program the mannequins, manage direction, create scenarios, etc.)
   - Organisational secretary
   - Healthcare personnel (doctors, postgraduates, nurses, etc.) who coordinate the simulation activities
   - Other (please specify)
2. What is the source of funding for the simulation programme at your residency?
   - Hospital
   - Postgraduate school/Pediatrics division
   - University
   - Public funds
   - Pharmaceutical companies
   - Private organisations/philanthropic associations
   - Not sure
   - No funds are available
   - Other (please specify)
3. Have you had the opportunity to participate in simulation-based courses organized outside your residency?
   - Yes
   - No
4. What are the main barriers to participation in simulation-based training outside your residency (indicate all which apply)?
   - Costs associated with “external” courses and related transfer (travel, accommodations, etc.)
   - Work scheduling issues due to lack of coverage for postgraduates participating in training
   - Simulation-based courses are not available nearby
   - My school is able to provide analogous courses without the necessity of transfer
   - Other (please specify)

***Indicate your response (indicate all which apply) for each of the following procedures.***

***The use of low and medium fidelity mannequins is also included.***

1. Bag Valve Mask ventilation :
   - Procedure is currently taught with simulation.
   - Postgraduate can put this procedure into practice on patients after a fixed number of simulations.
   - Procedure is taught directly on patients.
   - Postgraduate is required to conduct a fixed number of this procedure on patients by the end of the year of specialization.
   - The postgraduate’s ability to perform this procedure is evaluated by other doctors.
   - This procedure is not taught.
   - The postgraduate’s ability to perform this procedure is not evaluated.
   - Other (please specify)

1. Endotracheal intubation:
   - Procedure is currently taught with simulation.
   - Postgraduate can put this procedure into practice on patients after a fixed number of simulations.
   - Procedure is taught directly on patients.
   - Postgraduate is required to conduct a fixed number of this procedure on patients by the end of the year of specialization.
   - The postgraduate’s ability to perform this procedure is evaluated by other doctors.
   - This procedure is not taught.
   - The postgraduate’s ability to perform this procedure is not evaluated.
   - Other (please specify)
2. Difficult airway management:
   - Procedure is currently taught with simulation.
   - Postgraduate can put this procedure into practice on patients after a fixed number of simulations.
   - Procedure is taught directly on patients.
   - Postgraduate is required to conduct a fixed number of this procedure on patients by the end of the year of specialization.
   - The postgraduate’s ability to perform this procedure is evaluated by other doctors.
   - This procedure is not taught.
   - The postgraduate’s ability to perform this procedure is not evaluated.
   - Other (please specify)
3. Cardioversion/defibrillation:
   - Procedure is currently taught with simulation.
   - Postgraduate can put this procedure into practice on patients after a fixed number of simulations.
   - Procedure is taught directly on patients.
   - Postgraduate is required to conduct a fixed number of this procedure on patients by the end of the year of specialization.
   - The postgraduate’s ability to perform this procedure is evaluated by other doctors.
   - This procedure is not taught.
   - The postgraduate’s ability to perform this procedure is not evaluated.
   - Other (please specify)
4. Cardiopulmonary resuscitation:
   - Procedure is currently taught with simulation.
   - Postgraduate can put this procedure into practice on patients after a fixed number of simulations.
   - Procedure is taught directly on patients.
   - Postgraduate is required to conduct a fixed number of this procedure on patients by the end of the year of specialization.
   - The postgraduate’s ability to perform this procedure is evaluated by other doctors.
   - This procedure is not taught.
   - The postgraduate’s ability to perform this procedure is not evaluated.
   - Other (please specify)
5. Central venous access:
   - Procedure is currently taught with simulation.
   - Postgraduate can put this procedure into practice on patients after a fixed number of simulations.
   - Procedure is taught directly on patients.
   - Postgraduate is required to conduct a fixed number of this procedure on patients by the end of the year of specialization.
   - The postgraduate’s ability to perform this procedure is evaluated by other doctors.
   - This procedure is not taught.
   - The postgraduate’s ability to perform this procedure is not evaluated.
   - Other (please specify)

………………………………………………………………………………………………

1. Umbilical venous access:
   - Procedure is currently taught with simulation.
   - Postgraduate can put this procedure into practice on patients after a fixed number of simulations.
   - Procedure is taught directly on patients.
   - Postgraduate is required to conduct a fixed number of this procedure on patients by the end of the year of specialization.
   - The postgraduate’s ability to perform this procedure is evaluated by other doctors.
   - This procedure is not taught.
   - The postgraduate’s ability to perform this procedure is not evaluated.
   - Other (please specify)

………………………………………………………………………………………………

1. Intraosseous access:
   - Procedure is currently taught with simulation.
   - Postgraduate can put this procedure into practice on patients after a fixed number of simulations.
   - Procedure is taught directly on patients.
   - Postgraduate is required to conduct a fixed number of this procedure on patients by the end of the year of specialization.
   - The postgraduate’s ability to perform this procedure is evaluated by other doctors.
   - This procedure is not taught.
   - The postgraduate’s ability to perform this procedure is not evaluated.
   - Other (please specify)

………………………………………………………………………………………………

1. Lumbar puncture:
   - Procedure is currently taught with simulation.
   - Postgraduate can put this procedure into practice on patients after a fixed number of simulations.
   - Procedure is taught directly on patients.
   - Postgraduate is required to conduct a fixed number of this procedure on patients by the end of the year of specialization.
   - The postgraduate’s ability to perform this procedure is evaluated by other doctors.
   - This procedure is not taught.
   - The postgraduate’s ability to perform this procedure is not evaluated.
   - Other (please specify)
   - Other (please specify)

……………………………………………………………………………

1. Positioning of thoracic drainage:
   - Procedure is currently taught with simulation.
   - Postgraduate can put this procedure into practice on patients after a fixed number of simulations.
   - Procedure is taught directly on patients.
   - Postgraduate is required to conduct a fixed number of this procedure on patients by the end of the year of specialization.
   - The postgraduate’s ability to perform this procedure is evaluated by other doctors.
   - This procedure is not taught.
   - The postgraduate’s ability to perform this procedure is not evaluated.
   - Other (please specify)
